# New Conjugatable Platinum(II) Chlorins: Synthesis, Reactivity and Singlet Oxygen Generation

**DOI:** 10.3390/molecules30122496

**Published:** 2025-06-06

**Authors:** José Almeida, Giampaolo Barone, Luís Cunha-Silva, Ana F. R. Cerqueira, Augusto C. Tomé, Maria Rangel, Ana M. G. Silva

**Affiliations:** 1 LAQV-REQUIMTE, Departamento de Química e Bioquímica, Faculdade de Ciências da Universidade do Porto, 4169-007 Porto, Portugal; l.cunha.silva@fc.up.pt; 2 Dipartimento di Scienze e Tecnologie Biologiche, Chimiche e Farmaceutiche, Università di Palermo, Viale delle Scienze, Edificio 17, 90128 Palermo, Italy; giampaolo.barone@unipa.it; 3 LAQV-REQUIMTE, Department of Chemistry, University of Aveiro, 3810-193 Aveiro, Portugal; anacerqueira@ua.pt (A.F.R.C.); actome@ua.pt (A.C.T.); 4 LAQV-REQUIMTE, Instituto de Ciências Biomédicas Abel Salazar, Universidade do Porto, 4050-313 Porto, Portugal; mrangel@icbas.up.pt

**Keywords:** chlorins, metallochlorins, platinum(II) complexes, microwave-mediated metallation, optical properties, singlet oxygen generation

## Abstract

An efficient protocol was developed for the microwave-mediated metallation of 5-(4-methoxycarbonylphenyl)-10,15,20-tris(pentafluorophenyl)porphyrin (**P1**) with bis(benzonitrile)platinum dichloride salt and subsequent 1,3-dipolar cycloaddition of the resulting **PtP1** with an azomethine ylide to give two isomeric metallochlorins: **PtC1** (main isomer) and **PtC3**. The methyl ester group of metalloporphyrin **PtP1** and metallochlorin **PtC1** was successfully hydrolysed in an alkaline medium to yield the corresponding derivatives **PtP2** and **PtC2** in moderate-to-good yields. As a proof of concept of the reactivity of the carboxy group in **PtP2** and **PtC2**, these compounds were conjugated with a hydroxylated derivative of indomethacin, a known potent non-steroidal anti-inflammatory, obtaining the conjugates **PtP2**-**Ind** and **PtC2**-**Ind**. The obtained platinum(II) porphyrins and chlorins were characterized by UV-Vis, NMR spectroscopy and mass spectrometry. The structure of **PtP1** was also confirmed by X-ray crystallography. Singlet oxygen generation studies were carried out, as well as theoretical calculations, which demonstrated that the prepared Pt(II) complexes can be considered potential photosensitizers for PDT.

## 1. Introduction

Photodynamic therapy (PDT) is a therapeutic approach that integrates three essential components, light, molecular oxygen and a photosensitizer, to generate reactive oxygen species (ROS), capable of inducing cellular damage [1]. This modality has diverse applications across various medical fields, including the treatment of various cancers, such as head, neck, esophageal, lung, bladder, colorectal, prostate and skin cancers [2,3,4,5]. Additionally, PDT is gaining prominence in combating infectious diseases, due to its antimicrobial properties, particularly against drug-resistant bacteria and other pathogens, a field referred to as antimicrobial photodynamic therapy (aPDT) [6,7,8].

**Figure 1:**
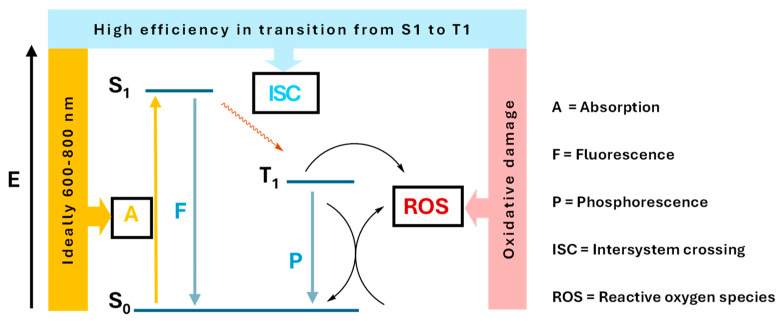
Critical parameters for enhanced photodynamic activity (where E = energy and S_0_ = ground state).

Recent advancements in PDT have focused on the development of new photosensitizers and genetic engineering of biological photosensitizers to enhance ROS production and study cellular signalling pathways [9]. To achieve maximum therapeutic efficiency with minimal side effects, three critical features of an ideal photosensitizer should be met (Figure 1): (1) it should preferentially accumulate in the target tissue or cells, minimizing damage to healthy surrounding areas; (2) ideally, it should absorb light within the “therapeutic window” (600–800 nm), where tissue penetration is optimal; and (3) it should exhibit efficient intersystem crossing (ISC) from its singlet excited state (S1) to its triplet excited state (T1). This transition is crucial for generating reactive oxygen species, particularly singlet oxygen (^1^O_2_), which serves as the primary cytotoxic agent in the PDT process.

Among recent advances in photosensitizers, platinum(II) complexes of porphyrins have received significant attention for their potential in PDT. Notably, Pt(II) metallochlorins exhibit exceptional phototoxicity against cancer cell lines such as HeLa and A375, highlighting their promise as effective photosensitizers for therapeutic applications [10,11,12]. The structural features of platinum(II) porphyrin complexes play a pivotal role in their efficacy as photosensitizers. Crystallographic studies indicate that the bond length between platinum(II) and nitrogen atoms is approximately 2.00 Å, ensuring an optimal fit within the porphyrin core. This configuration preserves the molecule’s planarity, a critical attribute for enhancing singlet oxygen generation via the heavy-atom effect. This effect promotes ISC between singlet (S1) and triplet (T1) excited states, thereby increasing the photosensitizer’s therapeutic efficiency (Figure 1) [10].

Moreover, these platinum complexes exhibit strong phosphorescence, opening avenues for their application in cancer photodiagnosis. This dual functionality—therapeutic and diagnostic—positions platinum(II) porphyrin complexes as highly valuable agents in cancer research and treatment, offering potential for both effective therapy and real-time diagnostic imaging [11].

The present study aims to contribute to advancements in PDT by establishing an efficient methodology for the metallation of porphyrin **P1** with platinum(II), its transformation into platinum(II) chlorins through 1,3-dipolar cycloaddition (1,3-DC) and the hydrolysis of the methyl ester to the more hydrophilic carboxylate. This approach aims to improve the stability and functionality of these compounds as photosensitizers. The reactivity of the carboxylate group is further investigated by conjugating the resulting Pt(II) complexes with an indomethacin derivative, potentially yielding novel dual-functional therapeutic agents. Additionally, the optical properties of these compounds are determined by UV-Vis spectroscopy, which is critical for their performance in PDT. One of the platinum(II) complexes, **PtP1**, is analyzed by X-ray crystallography studies, allowing us to obtain additional structural data on this type of complex. Finally, the photosensitizer activity is assessed by calculating the triplet excited state, offering insights into their capacity to generate singlet oxygen for therapeutic applications.

## 2. Results and Discussion

### 2.1. Synthesis

**Scheme 1:**
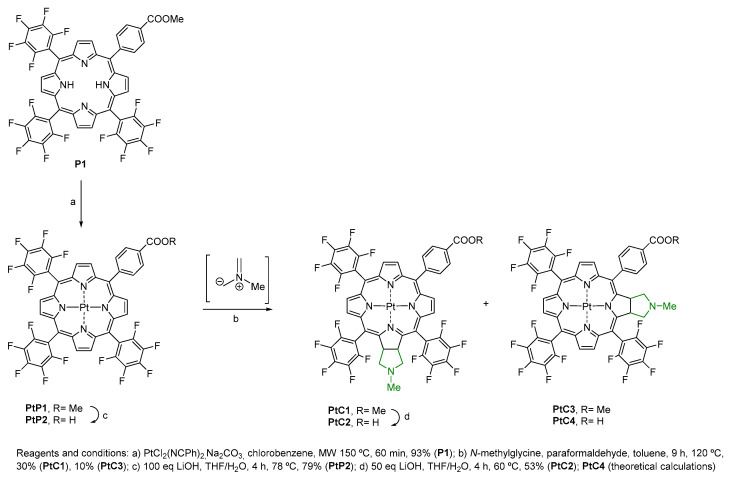
Reaction pathway for obtaining platinum(II) complexes of porphyrins (**PtP1** and **PtP2**) and pyrrolidine-fused chlorins (**PtC1**, **PtC2** and **PtC3**).

A. Synthesis of Pt(II) metalloporphyrin **PtP1**. The synthesis of Pt(II) porphyrin complexes presents several challenges. As a starting point, we used a microwave-mediated protocol, commonly reported in the literature, which involved heating a solution of porphyrin in benzonitrile with an excess of platinum(II) chloride (PtCl_2_) [11,12]. However, during the complexation of porphyrin **P1** (Scheme 1), degradation products, including a porphyrin lacking the COOMe group (Krapcho-type decarboxylation), were observed. To address this issue and optimize the metallation yield, several modifications to the synthetic procedure were explored (Table 1):

(1) Replacing PtCl_2_ with a platinum salt less susceptible to oxidation (K_2_PtCl_4_). However, there was no better progression of the reaction, even increasing the reaction time to 2 h (entries 1–5, Table 1).
(2) Verifying the release of HCl in the reaction mixture with PtCl_2_—we decided to use a base (Na_2_CO_3_) to reduce the temperature and increase the reaction time. However, a complete conversion of the porphyrin into the platinum(II) complex was not observed (entry 6, Table 1).
(3) The use of a platinum salt soluble in organic solvents—bis(benzonitrile)platinum(II) dichloride—significantly improved the yield of complex **PtP1**. In this case, it was possible to replace benzonitrile with chlorobenzene as a solvent with a lower boiling point, facilitating the distillation step at the end of the reaction. Using these conditions in the presence of a base (NaOAc or Na_2_CO_3_), at a lower temperature (150 °C), it was possible to isolate platinum complex **PtP1** (Scheme 1) in satisfactory yield (entries 7–8, Table 1).

**Table 1:** Optimization of the reaction conditions for the microwave-mediated synthesis of platinum(II) porphyrin complex **PtP1**.

Entry	Pt(II) Salt (Equiv.)	Solvent	Base (Equiv.)	P1 (mol/dm^3^)	Temp. (°C)	Time (min)	PtP1 (%)
1	K_2_PtCl_4_ (3)	benzonitrile	-	0.005	250	20	Mixture with **P1**
2	K_2_PtCl_4_ (3)	benzonitrile	-	0.004	250	20	Mixture with **P1**
3	K_2_PtCl_4_ (3)	benzonitrile	-	0.004	(1) 180; (2) 200	20 + 20	Mixture with **P1**
4	K_2_PtCl_4_ (3)	benzonitrile	-	0.006	230	20	Mixture with **P1**
5	K_2_PtCl_4_ (3)	benzonitrile	-	0.005	250	120	Mixture with **P1**
6	PtCl_2_ (3)	benzonitrile	Na_2_CO_3_ (19)	0.011	150	60 + 120	Mixture with **P1**
7	PtCl_2_(PhCN)_2_ (2)	chlorobenzene	NaOAc (5)	0.041	150	60 + 60	83
8	PtCl_2_(PhCN)_2_ (2)	chlorobenzene	Na_2_CO_3_ (5)	0.041	150	60 + 60	93

B. 1,3-Dipolar cycloaddition reaction. With metalloporphyrin **PtP1** in hand, the subsequent 1,3-DC reaction was performed using an azomethine ylide generated in situ from sarcosine and paraformaldehyde (Scheme 1). This reaction yielded a mixture of two isomeric metallochlorins, **PtC1** and its regioisomer **PtC3**, which were effectively separated through chromatographic purification. The major product, **PtC1**, was obtained in a 30% yield, while the minor regioisomer, **PtC3**, was isolated in a 10% yield.

The formation of these two regioisomers was anticipated, since starting porphyrin **PtP1** contains two non-equivalent reactive sites where the cycloaddition can occur. Computational calculations were performed on the hydrolysed forms **PtC2** and **PtC4**, before moving on to further functionalization reactions, in order to infer their stability. Density functional theory (DFT) calculations revealed that the **PtC2** isomer is more stable by 1.5 kJ/mol than **PtC4** (Figure S16), aligning well with the experimental results, where **PtC1** was the major adduct. Further studies were focused on this isomer. The M06-L functional was chosen in our computational model, since it has been proven that it is able to provide reliable structural and energetic results for metal compounds [13,14,15], as also recently verified by us [16,17,18].

C. Ester hydrolysis. The methyl ester groups of metalloporphyrin **PtP1** and metallochlorin **PtC1** were successfully hydrolysed using a lithium hydroxide (LiOH) solution in a THF/H_2_O mixture. This reaction yielded the corresponding carboxylate derivatives, **PtP2** and **PtC2**, in 79% and 53% yields, respectively (Scheme 1).

Under these conditions, the integrity of the macrocycles was fully preserved, as evidenced by the following observations: (i) the fluorine atoms at the *para*-position of the phenyl rings remained there (there is no evidence of substitution reactions); (ii) the metal ion complexes were stable, with no signs of metal removal; and (iii) the carboxy group was retained (i.e., no decarboxylation occurred). This method effectively provided the desired metalloporphyrin and metallochlorin carboxylic acids while maintaining the structural and chemical stability of the macrocyclic framework.

D. Conjugation with indomethacin derivative. Indomethacin, a non-steroidal anti-inflammatory drug (NSAID), has been investigated in combination with photosensitizers to enhance the efficacy of photodynamic therapy (PDT) [19,20,21]. Previously, our group synthesized porphyrin–indomethacin and chlorin–indomethacin conjugates, which demonstrated high singlet oxygen generation for both the chlorin and its conjugate, as well as significant cellular uptake of the chlorin–indomethacin conjugate, with localization, as small aggregates, in cancer cells [22].

**Scheme 2:**
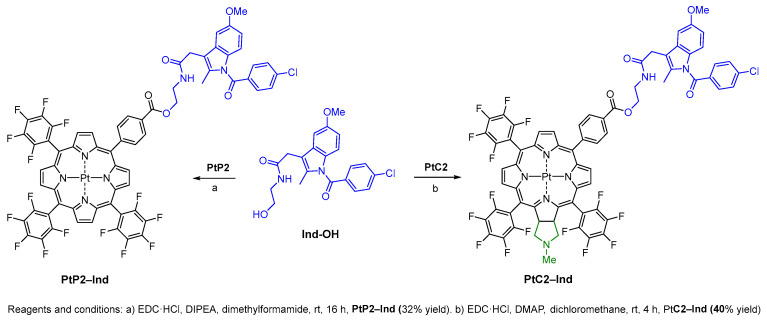
Synthetic procedure to obtain **PtP2**-**Ind** and **PtC2**-**Ind**.

Based on these findings, and as a proof of concept for the reactivity of the carboxy groups in **PtP2** and **PtC2**, we promoted their esterification with a hydroxyalkyl derivative of indomethacin (Ind-OH). The expected **PtP2**-**Ind** and **PtC2**-**Ind** conjugates were obtained in moderate yields (Scheme 2).

The reaction of Ind-OH with **PtP2** proceeded efficiently using EDC·HCl as a coupling agent and DIPEA as a base, producing the **PtP2**-**Ind** conjugate in a 32% yield. However, the conjugation with **PtC2** proved more challenging, requiring several optimizations. Ultimately, the use of EDC·HCl and a catalytic amount of DMAP in dichloromethane at room temperature for 4 h successfully yielded **PtC2**-**Ind** in a 40% yield. It should be noted that the **PtC2**-**Ind** conjugate showed some instability in solution, resulting in the formation of multiple spots observed on thin-layer chromatography (TLC). The degradation of this compound in solution may be related to the oxidation of the pyrrolidine ring.

Minimum energy conformations were determined by DFT calculations for the porphyrin–indomethacin and chlorin–indomethacin conjugates **PtP2**-**Ind** and **PtC2**-**Ind** (Figure S17), revealing that the most stable conformations involve an intramolecular π–π stacking between the parallel-displaced indomethacin moiety and the *meso*-phenyl ring of the macrocycle. This indomethacin-stacked conformation is in accordance with results reported previously for similar conjugates [22].

### 2.2. Characterization of Compounds

The compounds were characterized by NMR spectroscopy (Figures S2–S12) and mass spectrometry (Figures S13–S15).

**Figure 2:**
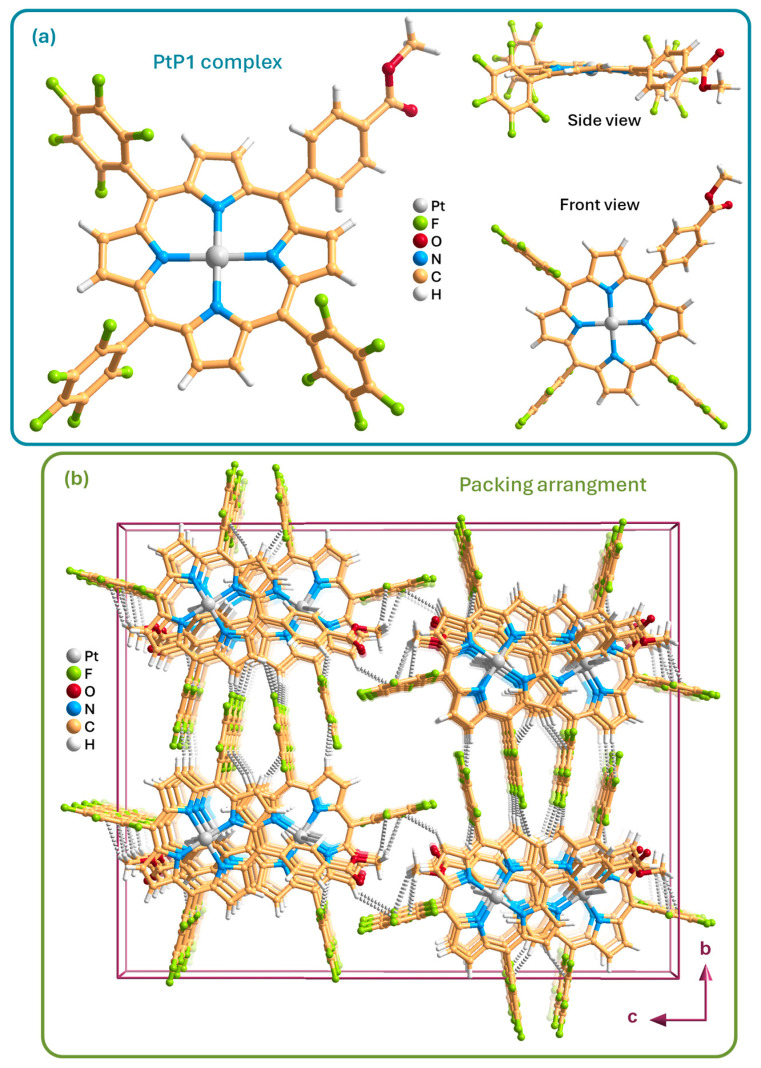
Details of the X-ray diffraction structure of the metalloporphyrin **PtP1**: (**a**) different perspective views of the individual complex and (**b**) extended packing arrangement (with the weak hydrogen weak bonds C−H···F shown as grey dashed lines).

A. Crystal structure of **PtP1**. Crystalline material of metalloporphyrin **PtP1** of sufficient quality for single-crystal X-ray diffraction analysis was obtained by recrystallization from dichloromethane/hexane. The crystalline structure crystallized in the monoclinic system and C2/c space group (Figure 2a), with the asymmetric unit comprising a unique metalloporphyrin molecule. While the crystallographic structure previously reported for [*meso*-tetrakis(pentafluorophenyl)porphyrinato]platinum(II) (PtTPPF_20_) [23] reveals a nearly planar porphyrin core with essentially orthogonal C_6_F_5_ rings, the structure of **PtP1** herein reported shows a porphyrin core with evident bending (Figure 2a—side view). In addition, the Pt−N distances (Pt1−N3, 2.008(2) Å; Pt1−N4, 2.011(2) Å; Pt1−N1, 2.014(2) Å; and Pt1−N3, 2.018(2) Å) in this new metalloporphyrin are comparable to those of the PtTPPF_20_. Metalloporphyrin **PtP1** is involved in an extended network of weak hydrogen bonds, namely C−H···F (represented as grey dashed bonds in Figure 2b), leading to global packing with spaces (channels and pockets) occupied by solvent molecules. A brief pore analysis of the voids, using MERCURY software (v4.2.0), revealed continuous pores along the *a*-axis (or [1 0 0] direction), with crystallographic inversion centres in the middle (the minimum accessible pore diameter is 3.8 Å and maximum diameter is 5.2 Å). The solvent molecules, most probably hexane and residual water, are not shown, since they are structurally disordered and impossible to accurately locate, model and refine.

B. UV-Vis spectroscopy. The UV-Vis absorption spectra of the compounds were assessed in dimethylformamide (DMF) solutions (Figure S1). Table 2 presents the spectral data obtained for the platinum(II) complexes, which are compared with their respective free-metal bases, **P2** and **C2**. The introduction of the platinum(II) metal ion into the porphyrin ring results in a significant hypsochromic shift in the Soret and Q bands. The two isomeric metallochlorins **PtC1** and **PtC3** exhibit absorption spectra with a notable hypsochromic shift in the Q bands, exhibiting a maximum absorption band at 591 nm, for both compounds. This indicates that these metallochlorins retain a PDT-applicable absorption around 600 nm. Additionally, conjugation with indomethacin does not result in significant alterations to the absorption properties of the macrocycles.

By comparing the UV-Vis absorption spectra of our platinum(II) chlorin complexes in DMF with reported data for a similar platinum(II) chlorin in CHCl_3_ (Soret band at 395 nm and Q-bands in the 478–596 nm region) [10], we conclude that there are no significant differences in the absorption wavelengths. This indicates that DMF does not induce significant changes in the fundamental UV-Vis absorption profiles of the platinum(II) chlorins studied.

**Figure 3:**
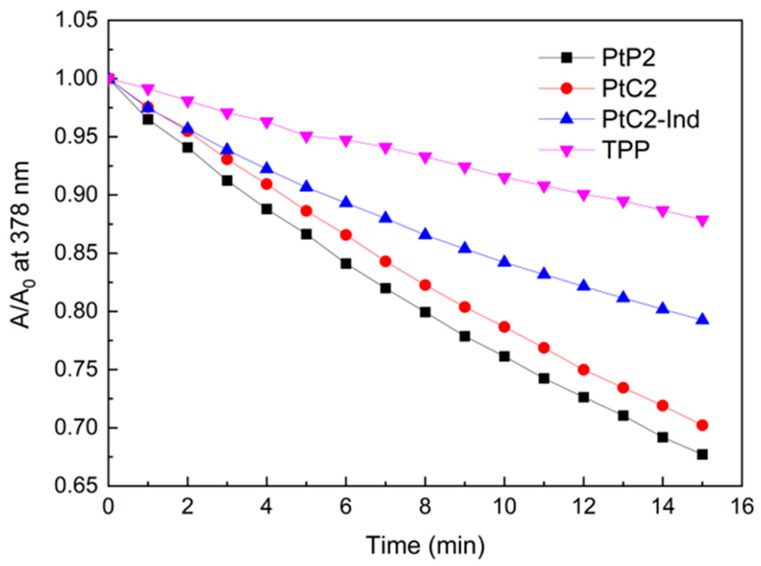
Photooxidation of 9,10-dimethylanthracene (DMA) sensitized by **PtP2**, **PtC2**, **PtC2**-**Ind** and 5,10,15,20-tetraphenylporphyrin (**TPP**) used as a standard. Solvent: DMF.

C. Singlet oxygen generation. To further investigate the photosensitizing activity of the synthesized compounds, their capability to generate singlet oxygen (^1^O_2_) was evaluated by monitoring the photooxidation of 9,10-dimethylanthracene (DMA). The samples were irradiated at 420 nm under aerobic conditions, and the photooxidation of DMA was monitored by the decrease in absorbance at λ_max_ = 378 nm (Figure 3). ^1^O_2_ quantum yields (ΦΔ) were determined to be 0.87, 0.90 and 0.65 for **PtP2**, **PtC2** and **PtC2**-**Ind**, respectively, with 5,10,15,20-tetraphenylporphyrin (**TPP**) (ΦΔ = 0.65) as a standard (*see infra* and SI for ^1^O_2_ quantum yield details).

Both **PtP2** and **PtC2** were identified as excellent generators of ^1^O_2_. On the other hand, the conjugate **PtC2**-**Ind**, obtained through the esterification of the carboxy group of **PtC2** with the **Ind**-**OH** derivative, exhibits increased lipophilicity compared to **PtP2** and **PtC2**. We believe that this alteration in its lipophilicity enhances intermolecular interactions, such as van der Waals forces and π−π stacking, which can lead to significant aggregation phenomena in solution and potentially quench ^1^O_2_ formation.

By comparing the conjugate **PtC2**-**Ind** and **TPP**, although their calculated ΦΔ values are comparable (both ~0.65), **PtC2**-**Ind** demonstrates a faster rate of DMA degradation, as observed in the steeper decay in absorbance over time (Figure 3). This difference in apparent photoactivity may arise from factors such as solubility, aggregation state or interaction with DMA, which can affect the effective rate of singlet oxygen transfer in solution beyond the intrinsic ΦΔ.

D. Triplet excited state calculations. Considering the good results obtained for the singlet oxygen generation of **PtP2** and **PtC2**, and to better understand the photosensitizing capacity of these compounds, the structures and energies of the first triplet excited states were calculated. We also included the minor isomer **PtC4** to evaluate the influence of the position of the pyrrolidine ring in the photosensitizing activity of these chlorins.

The results (Table 3) indicate that, although the structures of the triplet states are essentially identical to those of the singlet states, the large energy differences observed are essentially attributable only to the change in the singlet to triplet spin states. Interestingly, the triplet state energies of **PtC2** and **PtC4** are negligible, meaning that the position of the pyrrolidine ring does not significantly influence the efficiency of the triplet state. Furthermore, the energy differences obtained were significantly above the optimal threshold of 91.2 kJ/mol, the experimental value required for singlet oxygen production, supporting the potential photosensitizing activity of these complexes [24,25].

## 3. Experimental Section

### 3.1. Materials and Methods

Reagents and solvents purchased were reagent-grade and used without further purification unless otherwise stated. Bis(benzonitrile)dichloroplatinum(II) [26] and 5-(4-methoxycarbonylphenyl)-10,15,20-tris(pentafluorophenyl)porphyrin (**P1**) [27] were synthesized as previously described. Indomethacin derivative (**Ind**-**OH**) was prepared from the amidation reaction of indomethacin, following the protocol published elsewhere [22].

Flash chromatography was accomplished using silica gel (Merck, Rahway, NJ, USA, 230–400 mesh), while preparative thin-layer chromatography (TLC) was performed on 20 × 20 cm glass plates coated with silica gel (Merck 60, 1 mm thick). In the case of analytical TLC, this was performed on precoated sheets with silica gel (Merck 60, 0.2 mm thickness).

Microwave-mediated metallations were carried out in a CEM Discovery Labmate circular single-mode cavity instrument (300 W max magnetron power output) from the CEM Holdings Corporation (Matthews, NC, USA). Reactions were performed in closed-vessel conditions.

High-resolution mass spectrometry (HRMS) analysis was executed by electrospray ionization (ESI) using the LTQ-Orbitrap-XL instrument (Thermo Scientific, Waltham, MA, USA) with the following ESI source parameters: electrospray needle voltage of 3.1 kV, nitrogen sheath gas set to 6, capillary temperature at 275 °C, capillary voltage of 41 V, and tube lens voltage of 130 V. Ionization polarity was adjusted according to sample. For the acquisition of MALDI-TOF spectra, a Bruker UltrafleXtreme MALDI-TOF/TOF mass spectrometer (Madison, WI, USA) equipped with a nitrogen laser was used. The samples were dissolved in acetone and mixed in a 1:1 ratio with the matrix preparation before being applied to the MALDI target plate. The matrix preparation was 5 mg/mL (2*E*)-2-cyano-3-(4-hydroxphenyl)prop-2-enoic acid, 50% (*v*/*v*) methanol, and 0.1% (*v*/*v*) trifluoroacetic acid (TFA) in water. Samples were analyzed in the reflector positive-ion mode for the *m*/*z* range between 600–3500.

Nuclear magnetic resonance (NMR) spectra for all compounds were recorded on a 400 MHz NMR spectrometer (operating at 400.15 MHz for protons and 376.46 MHz for fluorine atoms), where CDCl_3_, or DMSO-d*_6_* were used as solvents and TMS as the internal reference. The chemical shifts (δ) are expressed in ppm and the coupling constants (*J*) in Hz. In the case of ^19^F NMR spectra, C_6_H_5_CF_3_ was used as a reference.

Electronic absorption spectra were recorded on a Shimadzu UV-3600 UV-Vis-NIR spectrophotometer (Kyoto, Japan) equipped with Shimadzu TCC-Controller (TCC-240A), at 25 °C, in 1.00 cm cuvettes, in the wavelength range of 300–800 nm. The stock solutions were prepared in DMF in concentration ranges of 10^−5^–10^−7^ M for the determination of the molar absorptivity coefficient (ε).

Crystalline material of metalloporphyrin **PtP1** was collected from the crystallization vial, immediately immersed in highly viscous, suitable single crystal mounted on a CryoLoop with the assistance of a stereomicroscope [28]. Diffraction data were collected on a Bruker D8 diffractometer (Mo K_α_ graphite-monochromated radiation, λ = 0.71073 Å; equipped with a Photon II CPAD detector) with the acquisition controlled by the APEX2 software package (v24.2) [28]. The temperature of acquisition, 150(2) K, was set up with a cryosystem by the Oxford Cryosystems Series 700 (Oxford Cryosystems, Long Hanborough, UK). Images were processed with the software SAINT+ (v7.12a) [29], and absorption effects were corrected with the multi-scan method implemented in SADABS (v2.05) [30]. The structures were solved using the algorithm implemented in SHELXT-2014 [31,32] and refined by successive full-matrix least-squares cycles on *F*^2^ using the SHELXL-v.2014 [32,33]. All the non-hydrogen atoms were successfully refined using anisotropic displacement parameters, and H-atoms bonded to carbon were placed at their idealized positions using appropriate *HFIX* instructions in SHELXL. All these atoms were included in subsequent refinement cycles in riding-motion approximation with isotropic thermal displacement parameters (*U*_iso_) fixed at 1.2 or 1.5 × *U_eq_* of the relative atom. Some electron density was found on the data of the crystal structure, most probably due to additional disordered solvent molecules occupying the spaces created by the packing arrangement of metalloporphyrin. Efforts to accurately locate, model and refine these residues were ineffective, and the investigation for the total potential solvent area using the software package PLATON (v1.17) [34,35] confirmed the existence of cavities with potential solvent accessible void volume. Thus, the original data sets were treated with the programme SQUEEZE (v241123) [36]. Table S2 summarizes selected information about the crystal, the single-crystal X-ray data collection and the structure refinement. A CIF file has been deposited with the Cambridge Crystallographic Data Centre (CCDC) as supplementary publication data, CCDC2439121. Copies of the data can be obtained online from the CCDC website.

### 3.2. Synthesis of **PtP1**

**P1** (50 mg, 0.53 µmol) and chlorobenzene (2 mL) were transferred into a 10 mL thick-walled glass tube equipped with a teflon coated magnetic stir bar. The resulting solution was purged with nitrogen for 15 min. Afterwards, bis(benzonitrile)dichloroplatinum(II) (50 mg, 106 µmol) and sodium carbonate (150 mg, 1.83 mmol) were added to the solution and the vessel was sealed with a silicone septum and placed into the microwave cavity. The reaction mixture was then heated to 150 °C using a maximum microwave power of 250 W, which was automatically modulated for 1 h. After this time, one more portion of bis(benzonitrile)dichloroplatinum(II) (50 mg, 106 µmol) was added and the solution was heated as previously described for another 1 h. The resulting reaction mixture was filtered from an unidentified black powder, the chlorobenzene was evaporated, and the mixture was separated by silica gel column chromatography using toluene as an eluent to remove residual benzonitrile, and then a mixture of dichloromethane/hexane (12:8) was used to isolate the main reddish fraction, corresponding to **PtP1** (55 mg, 93% yield).

**PtP1**: ^1^H NMR (400.14 MHz, CDCl_3_) δ 4.12 (s, 2H, CO_2_Me), 8.22–8.29 (m, 2H, porphyrin-H-Ar), 8.42–8.52 (m, 2H, porphyrin-H-Ar), 8.74 (d, *J* = 5.2 Hz, 2H, H-β), 8.77–8.85 (m, 6H, H-β).

### 3.3. 1,3-DC Reaction of **PtP1** with Azomethine Ylide

*N*-methylglycine (140 mg, 1.57 mmol) and paraformaldehyde (23.5 mg, 0.783 mmol) were added to a solution of **PtP1** (178 mg, 0.157 mmol) in anhydrous toluene (18 mL) at 120 °C. The resulting mixture was maintained at 120 °C under a nitrogen atmosphere for 3 h. Two more additions of *N*-methylglycine and paraformaldehyde were performed until the completion of 9 h of reaction. After being cooled to room temperature, the reaction mixture was filtered to hold all solid residues. Afterwards, the filtrate was diluted in dichloromethane and washed twice with distilled water and brine. The organic phase was dried under anhydrous Na_2_SO_4_ and filtered, and the solvent was evaporated under vacuum. The resulting residue was dissolved in dichloromethane and the mixture was purified by flash chromatography (silica gel) using dichloromethane/acetone (99:1) as the eluent. After the recovery of the unreacted porphyrin **PtP1** (52.9 mg, 29.7% recovery), platinum(II) chlorin complex **PtC1** (57 mg, 30% yield) was eluted with dichloromethane/acetone (95:5) and the **PtC3** isomer (19 mg, 10% yield) with dichloromethane/acetone (90:10).

**PtC1**: ^1^H NMR (400.14 MHz, CDCl_3_) δ 2.25 (s, 3H, *N*-Me), 2.51–2.58 (m, 2H, CH_2_-pyrrolidine), 3.13–3.22 (m, 2H, CH_2_-pyrrolidine), 4.09 (s, 3H, CO_2_Me), 5.26–5.42 (m, 2H, H-2,3), 8.05–8.17 (m, 3H), 8.16–8.24 (m, 1H), 8.36–8.42 (m, 3H), 8.42–8.47 (m, 3H). UV-Vis (DMF) λ*_max_* (ε) 394 (178 × 10^3^); 476 (7.7 × 10^3^); 549 (10.7 × 10^3^); 591 (60 × 10^3^).

**PtC3**: ^1^H NMR (400.14 MHz, CDCl_3_) δ 2.16 (s, 3H, *N*-Me), 2.39–2.54 (m, 2H, CH_2_-pyrrolidine), 2.91–3.01 (m, 1H, CH_2_-pyrrolidine), 3.06–3.19 (m, 1H, CH_2_-pyrrolidine), 4.08 (s, 3H, CO_2_Me), 5.25–5.37 (m, 1H, H-17 or 18), 5.44–5.56 (m, 1H, H-17 or 18), 7.98–8.04 (m, 2H, H-Ar-porphyrin), 8.17–8.26 (m, 2H, H-Ar-porphyrin), 8.34–8.44 (m, 3H, H-β), 8.45–8.52 (m, 2H, H-β). UV-Vis (DMF) λ*_max_* (ε) 395 (186 × 10^3^); 482 (7.7 × 10^3^); 561 (15.8 × 10^3^); 598 (63 × 10^3^).

### 3.4. Ester Hydrolysis

#### 3.4.1. Synthesis of **PtP2**

A 50 mL round-bottomed flask containing a solution of **PtP1** (10.3 mg, 9.07 μmol) in a 1:1 mixture of THF and deionized water (10 mL) was placed in an oil bath at 78 °C followed by the addition of LiOH (22.9 mg, 0.956 mmol). The reaction occurred under reflux for 4 h. After this time, the THF was evaporated and a solution of HCl 1 mol/L was added dropwise until pH = 4. The extraction was performed with dichloromethane, and the organic phase was washed with water and brine, dried under anhydrous Na_2_SO_4_, filtered and then evaporated. The residue was redissolved in a minimum volume of dichloromethane and precipitated from hexane, obtaining 8.1 mg of **PtP2** (79% yield).

**PtP2**: ^1^H NMR (400.14 MHz, DMSO-*d*_6_) δ 8.36–8.42 (m, 4H, H-Ar-porphyrin), 8.89 (d, *J* = 5.2 Hz, 2H, H-β), 9.22 (d, *J* = 5.2 Hz, 2H, H-β), 9.30 (s, 4H, H-β), 13.34 (s, 1H, COOH). ^19^F{^1^H} NMR (376.46 MHz, DMSO-d6) δ −162.48 (ddd, *J* = 28.7, 16.3, 5.9 Hz, 6F, F*_meta_*-Ar), −153.80 (dt, *J* = 35.6, 22.3 Hz, 3F, F*_para_*-Ar), −139.42 (ddd, *J* = 32.3, 25.8, 7.5 Hz, 6F, F*_ortho_*-Ar). UV-Vis (DMF) λ*_max_* (ε) 394 (204 × 10^3^); 507 (17 × 10^3^); 539 (15 × 10^3^). MS(MALDI) *m/z*: 1122.157 [M + H]^+^, calcd. for [C_45_H_14_F_15_N_4_O_2_Pt]^+^ 1122.052.

#### 3.4.2. Synthesis of **PtC2**

A 50 mL round-bottomed flask containing a solution of **PtC1** (18.4 mg, 16.2 μmol) in a 1:1 mixture of THF and deionized water (18 mL) was placed in an oil bath at 78 °C followed by the addition of LiOH (37.7 mg, 1.57 mmol). The reaction occurred under reflux for 4 h. After this time, the THF was evaporated and a solution of HCl 1 mol/L was added dropwise until pH = 4. The extraction was performed with dichloromethane, and the organic phase was washed with water and brine, dried under anhydrous Na_2_SO_4_, filtered and then evaporated. The residue was redissolved in dichloromethane and purified by preparative TLC using dichloromethane/methanol (95:5) as the eluent, obtaining 5.8 mg (32% yield) of **PtC2**.

**PtC2**: ^1^H NMR (400.14 MHz, DMSO-*d_6_*) δ 2.10 (s, 3H, *N*-CH_3_), 2.36–2.45 (m, 2H, CH_2_-pyrrolidine, overlapped with the solvent signal), 3.17–3.26 (m, 2H, CH_2_-pyrrolidine, overlapped with residual water signal), 5.31–5.44 (m, 2H, H-2,3), 8.15–8.24 (m, 2H, -C_6_H_4_-), 8.32 (dq, 2H, *J* = 6.6 and 2.2 Hz, -C_6_H_4_-), 8.44 (dd, *J* = 5.1 and 2.1 Hz, 2H, H-β), 8.51 (dd, *J* = 5.1 and 1.4 Hz, 2H, H-β), 8.59 (dd, *J* = 5.2 and 1.5 Hz, 2 H, H-β), 8.77 (d, *J* = 5.0 Hz, 1H, H-β), 8.86 (d, *J* = 5.1 Hz, 1H, H-β). ^19^F{^1^H} NMR (376.46 MHz, DMSO-d6) δ −162.47 (qd, *J* = 26.3 and 7.5 Hz, 2F, F*_meta_*-Ar), −161.35 to −160.96 (m, 4F, F*_meta_*-Ar), −153.99 (t, *J* = 22.6 Hz, 1F, F*_para_*-Ar), −153.00 (td, *J* = 22.4 and 10.2 Hz, 2F, F*_para_*-Ar), −140.13 to −139.93 (m, 2F, F*_ortho_*-Ar), −139.47 to −139.23 (m, 2F, F*_ortho_*-Ar), −137.56 to −137.26 (m, 2F, F*_ortho_*-Ar). UV-Vis (DMF) λ*_max_* (ε) 394 (146 × 10^3^); 476 (6.5 × 10^3^); 549 (9.4 × 10^3^); 591 (52 × 10^3^). Fluorescence (deaerated DMF) 654 nm.

### 3.5. Conjugation with Indomethacin Derivative

#### 3.5.1. Synthesis of **PtP2**-**Ind**

EDC·HCl (0.0060 g, 0.031 mmol) was added to a pear-shaped 20 mL flask containing a solution of **PtP2** (0.035 g, 0.031 mmol) in DMF (3 mL), and this solution was left stirring under a nitrogen atmosphere for 10 min. After this time, DIPEA (20 µL, 0.0011 mmol) was added, followed by **Ind**-**OH** (0.013 g, 0.032 mmol). The resulting solution was left stirring overnight under a nitrogen atmosphere. The reaction mixture was then diluted with toluene to remove the DMF in the rotary evaporator. The residue was washed three times with distilled water, and the organic phase was dried with anhydrous Na_2_SO_4_, filtered, concentrated and purified by preparative thin-layer chromatography using a 96:4 mixture of dichloromethane/methanol as the eluent, obtaining 0.0151 g of **PtP2**-**Ind** (32% yield).

**PtP2**-**Ind**: ^1^H NMR (400.14 MHz, CDCl_3_) δ 2.46 (s, 3H, 2′-C*H*_3_), 3.72–3.77 (m, 4H, 4″-C*H*_2_, 1″-C*H*_2_), 3.80 (s, 3H, 5′-OC*H*_3_), 4.57 (t, 2H, *J =* 4.8 Hz, 5″-C*H*_2_), 6.22 (t, 1H, *J* = 5.6 Hz, 3″-N*H*), 6.66 (dd, 1H, *J* = 9.0 and 2.5 Hz, 6′-H-Ar), 6.81 (d, 1H, *J* = 9.0 Hz, 7′-H-Ar), 6.96 (d, 1H, *J* = 2.5 Hz, 4′-H-Ar), 7.27–7.35 (m, 2H, 10′, 14′-H-Ar), 7.58–7.68 (m, 2H, 11′, 13′-H-Ar), 8.08 (dd, 2H, *J* = 16.6 and 7.9 Hz, H-Ar-Porph), 8.17 (s, 4H, H-Ar-Porph), 8.75 (d, 2H, *J* = 5.1 Hz), 8.79–8.86 (m, 6H, H-β). ^19^F{^1^H} NMR (376.46 MHz; CDCl_3_) δ −161.20 (dt, 6F, *J* = 22.9 and 7.9 Hz, F*_meta_*-Ar), −151.31 (m, 3F), −136.60 to −136.46 (m, 4F, F*_ortho_*-Ar), −136.43 to −136.29 (m, 2F, F*_ortho_*-Ar). ^13^C{^1^H} NMR (100.63 MHz, CDCl_3_) δ: 13.4 (2′-*C*H_3_), 32.4 (C-1″), 39.6 (C-4″), 55.9 (5′-O*C*H_3_), 64.0 (C-5″), 100.7 (C-4′), 112.7 (C-6′), 115.3 (C-7′), 128.3 (C-Ar-Porph), 129.3 (C-11′ and C-13′), 129.8 (C-β), 130.8 (C-β), 132.8 (C-β), 134.1 (C-10′ and C-14′), 166.6 (*C*OO-Ind), 168.5 (C-8′), 170.4 (C-2″); UV-Vis (DMF) *λ_max_* (*ε*) 393 (275 × 10^3^); 507 (19 × 10^3^); 539 (19 × 10^3^) nm. HRMS (ESI) *m*/*z*: 1526.142 [M + Na]^+^; calcd for [C_66_H_32_ClF_15_N_6_NaO_5_Pt]^+^ 1526.147 (Δm = 3.3 ppm).

#### 3.5.2. Synthesis of **PtC2**-**Ind** Conjugate

**PtC2** (0.0202 g, 0.0171 mmol) was dissolved in anhydrous dichloromethane (0.8 mL), and the solution was stirred in an ice bath for 10 min. After this time, EDC·HCl (0.0042 g, 0.0219 mmol) was added, and the solution was left, stirring for another 10 min. Afterwards, a solution of **Ind**-**OH** (0.0073 g, 0.0182 mmol) and DMAP (catalytic quantity) in anhydrous dichloromethane (0.3 mL) was added dropwise to the **PtC2** solution, and it was left, stirring for 4 h. The reaction mixture was washed three times with deionized water, and the organic phase was dried with Na_2_SO_4_, filtered, concentrated and purified by preparative thin-layer chromatography using a 99:1 mixture of dichloromethane/methanol as the eluent, obtaining 0.0107 g of **PtC2**-**Ind** (40% yield).

**PtC2**-**Ind**: ^1^H NMR (400.14 MHz, CDCl_3_) 2.24 (s, 3H, pyrrolidine-C*H*_3_), 2.45 (s, 3H, 2′-C*H*_3_), 2.54 (q, 2H, *J* = 8.4 Hz, C*H*_2_-pyrrolidine), 3.16 (t, 2H, *J* = 7.5 Hz, C*H*_2_-pyrrolidine), 3.69–3.76 (m, 4H, 4″-C*H*_2_, 1″-C*H*_2_), 3.78 (s, 3H, 5′-OC*H*_3_), 4.53 (t, 2H, *J =* 5.2 Hz, 5″-C*H*_2_), 5.27–5.39 (m, 2H, H-12 and H-13), 6.19 (t, 1H, *J* = 5.5 Hz, 3″-N*H*), 6.64 (dd, 1H, *J* = 9.0 and *J* = 2.5 Hz, 6′-H-Ar), 6.79 (d, 1H, *J* = 9.0 Hz, 7′-H-Ar), 6.93 (d, 1H, *J* = 2.4 Hz, 4′-H-Ar), 7.31 (d, 2H, *J* = 8.7 Hz, 10′, 14′-H-Ar), 7.62 (d, 2H, *J* = 8.6 Hz, 11′, 13′-H-Ar), 7.99–8.04 (m, 2H), 8.05–8.13 (m, 4H), 8.19 (dd, 2H, *J* = 5.1 and *J* = 1.6 Hz), 8.38 (d, 1H, *J* = 5.0 Hz, H-β), 8.40–8.47 (m, 4H, H-β). UV-Vis (DMF) *λ_max_* 394; 476; 549; 591 nm. HRMS (ESI) *m*/*z*: 1561.2285 [M + H]^+^; calcd for [C_69_H_40_ClF_15_N_7_O_5_Pt]^+^ 1561.2183 (Δm = 6.5 ppm).

### 3.6. Singlet Oxygen Generation

To investigate the photosensitizing activity of the synthesized compounds, their capability to generate singlet oxygen was evaluated by monitoring the photo-oxidation of 9,10-dimethylanthracene (DMA), a singlet oxygen quencher. Solutions of each compound presented in Figure 3 (**PtP2**, **PtC2** and **PtC2**-**Ind**) and 5,10,15,20-tetraphenylporphyrin (**TPP**) (Φ_Δ_ = 0.65) [37,38] in DMF (2.5 mL) were prepared in quartz cells (Abs_420_ ≈ 0.1).

Subsequently, a 30 μM solution of DMA in DMF was added and the resulting solutions were irradiated with monochromatic light (λ = 420 nm). The absorbance decay of DMA at 378 nm was measured at intervals of 60 s over a period of 900 s, and the results were registered. The kinetics of DMA photooxidation in DMF, in the absence of any PS, was also assessed, and no significant photodegradation was observed under the same irradiation conditions.

### 3.7. DFT Calculations

The geometry of **PtC2**, **PtC4**, **PtP2** (Figure S16) and two conformations of **PtC2**-**Ind** and **PtP2**-**Ind** (Figure S17) was fully optimized by DFT calculations, using the M06-L [39] functional, the CEP-121G [40] effective core potential basis set for Pt and the 6–311G(d,p) [41] basis set for the lighter atoms, in both singlet and triplet spin states, as implemented in the Gaussian 16 programme package [42]. The effect of the DMF solvent was included using the PCM [43] implicit method. Vibrational frequency calculations, within the harmonic approximation, were performed to check that each optimized geometry corresponded to an energy minimum and to obtain the relative standard Gibbs free energy values in solution.

## 4. Conclusions

In conclusion, we successfully synthesized a novel platinum(II) porphyrin (**PtP1**) using an optimized microwave irradiation protocol. This metalloporphyrin was subsequently converted into chlorin derivatives, **PtC1**, **PtC2** and **PtC3**. The corresponding carboxylic acid derivatives were converted into platinum(II) porphyrin–indomethacin (**PtP2**-**Ind**) and platinum(II) chlorin–indomethacin (**PtC2**-**Ind**) conjugates through a standard esterification process with a hydroxyalkyl indomethacin derivative. Our findings indicate that the incorporation of indomethacin reduces the singlet oxygen generation capabilities of the **PtC2**-**Ind** conjugate, but, even so, it remains a better singlet oxygen generator than TPP. However, **PtP2** and **PtC2** showed high singlet oxygen generation ability. DFT calculations further support this observation, revealing that the energy difference between triplet and singlet states exceeds the optimal threshold of 91.2 kJ/mol necessary for effective singlet oxygen production. The preferred indomethacin-stacked conformation of both conjugates likely contributes to their reduced singlet oxygen generation, as it promotes aggregation.

These results highlight the intricate relationship between molecular structure and photophysical properties, paving the way for future studies on the optimization of metalloporphyrin-based systems for therapeutic applications.

## Figures and Tables

**Table 2:** Absorption data of porphyrins (**P2** and **PtP2**), chlorins (**C2**, **PtC1, PtC2** and **PtC3**) and indomethacin conjugates (**P2**-**Ind** and **PtC2**-**Ind**) in dimethylformamide at 25 °C.

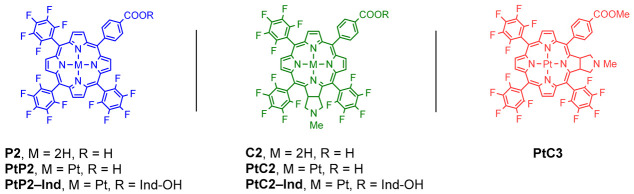					
	λ_max/nm_ (logε)				
	Soret Band	Q Bands			
**P2**	413 (5.44)	507 (4.23)	536 (3.46)	581 (3.73)	652 (3.35)
**PtP2**	394 (5.31)	507 (4.23)	539 (4.18)		
**C2**	408 (5.14)	503 (4.08)	530 (3.79)	594 (3.68)	648 (4.64)
**PtC1**	394 (5.25)	476 (3.89)	549 (4.03)	591 (4.78)	
**PtC2**	394 (5.16)	476 (3.81)	549 (3.97)	591 (4.72)	
**PtC3**	395 (5.27)	481 (4.03)	560 (4.19)	598 (4.80)	
**PtP2**-**Ind**	393 (5.44)	507 (4.29)	539 (4.29)		
**PtC2**-**Ind**	394	476	549	591	

**Table 3:** Energy differences between first excited triplet state and singlet ground state. The energy values are expressed in terms of relative standard Gibbs free energy calculated in DMF solution (see Figure S16).

**PtP2** triplet	+154.5 kJ/mol compared to the energy of the singlet.
**PtC2** triplet	+137.4 kJ/mol compared to the energy of the singlet.
**PtC4** triplet	+138.1 kJ/mol compared to the energy of the singlet.

## Data Availability

The data presented in this study are contained within the article and are also available in the Supplementary Materials.

## Supplementary Materials

The following supporting information can be downloaded at: https://www.mdpi.com/article/10.3390/molecules30122496/s1, Table S1: Structure, name, molecular formula and molecular weight for the synthetized macrocycles; Table S2: Crystal and structure refinement data for **PtP1** structure; Figure S1. Absorption spectra of the platinum complexes (**PtP2**, **PtP2**-**Ind**, **PtC1**, **PtC2**, **PtC3** and **PtC2**-**Ind**) in DMF; Figure S2. ^1^H NMR (400.14 MHz, CDCl_3_) spectrum of **PtP1**; Figure S3: ^1^H NMR (400.14 MHz, CDCl_3_) spectrum of **PtC1**; Figure S4: ^1^H NMR (400.14 MHz, CDCl_3_) spectrum of **PtC3**; Figure S5: ^1^H NMR (400.14 MHz, DMSO-d*_6_*) spectrum of **PtP2**; Figure S6: ^19^F NMR (376.46 MHz, DMSO-d*_6_*) spectrum of **PtP2**; Figure S7: ^1^H NMR (400.14 MHz, DMSO-d*_6_*) spectrum of **PtC2**; Figure S8: ^19^F NMR (376.46 MHz, DMSO-d*_6_*) spectrum of **PtC2**; Figure S9: ^1^H NMR (400.14 MHz, CDCl_3_) spectrum of **PtP2**-**Ind**; Figure S10: ^19^F NMR (376.46 MHz, CDCl_3_) spectrum of **PtP2**-**Ind**; Figure S11: ^13^C NMR (100.63 MHz, CDCl_3_) spectrum of **PtP2**-**Ind**; Figure S12: ^1^H NMR (400.14 MHz, CDCl_3_) spectrum of **PtC2**-**Ind**; Figure S13: MS (MALDI-TOF) spectrum of **PtP2**; Figure S14: MS (ESI) spectrum of **PtP2**-**Ind**; Figure S15: MS (ESI) spectrum of **PtC2**-**Ind**; Figure S16: Structures of **PtC2**, **PtC4** and **PtP2** obtained by full geometry optimization by DFT calculations; Figure S17: Structures of two conformations of **PtP2**-**Ind** and of **PtC2**-**Ind** obtained by full geometry optimization by DFT calculations.
