# Supplementary material for: New Conjugatable Platinum(II) Chlorins: Synthesis, Reactivity and Singlet Oxygen Generation
Source: Molecules. 2025 Jun 6;30(12):2496. doi: 10.3390/molecules30122496 (PMC12196018; doi:10.3390/molecules30122496)
Supplement: Supplementary file 1 [file molecules-30-02496-s001.zip › molecules-3654882-supplementary.pdf]

Supporting Information for

New Conjugatable Platinum(II) Chlorins: Synthesis, Reactivity and Singlet Oxygen Generation

José Almeida *, Giampaolo Barone, Luís Cunha-Silva, Ana F. R. Cerqueira, Augusto C. Tomé, Maria Rangel and Ana M. G. Silva *

Contents

1- Structures 2

1- Absorption spectra 3

2- NMR spectra 4

3- Mass spectra 9

4- DFT Calculations 11

5- X-ray 12

6- Singlet oxygen quantum yield details 12

7- References 13

1- Structures

Table S1. Structure, name, molecular formula and molecular weight for the synthetized macrocycles

Structure	Name	Molecular Formula	Molecular Weight
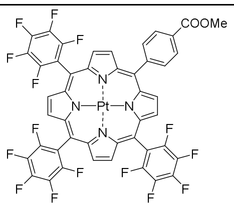	**PtP1**	$C_{46}H_{15}F_{15}N_4O_2Pt$	1135.7120
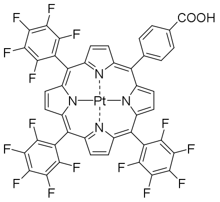	**PtP2**	$C_{45}H_{13}F_{15}N_4O_2Pt$	1121.6850
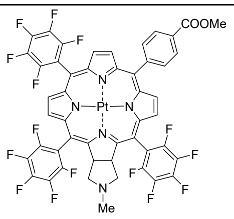	**PtC1**	$C_{49}H_{22}F_{15}N_5O_2Pt$	1192.8080
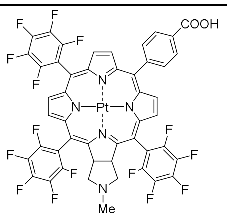	**PtC2**	$C_{48}H_{20}F_{15}N_5O_2Pt$	1178.7810
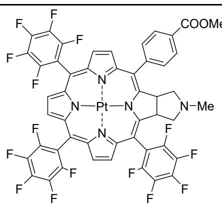	**PtC3**	$C_{49}H_{22}F_{15}N_5O_2Pt$	1192.8080
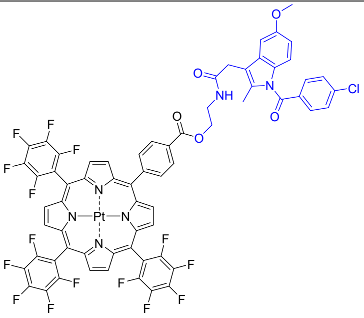	**PtP2-Ind**	$C_{66}H_{32}ClF_{15}N_6O_5Pt$	1504.5290

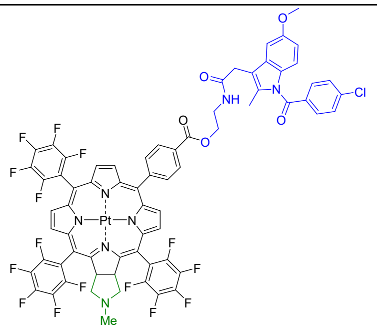	**PtC2-Ind**	$C_{69}H_{39}ClF_{15}N_7O_5Pt$	1561.6250

1- Absorption spectra

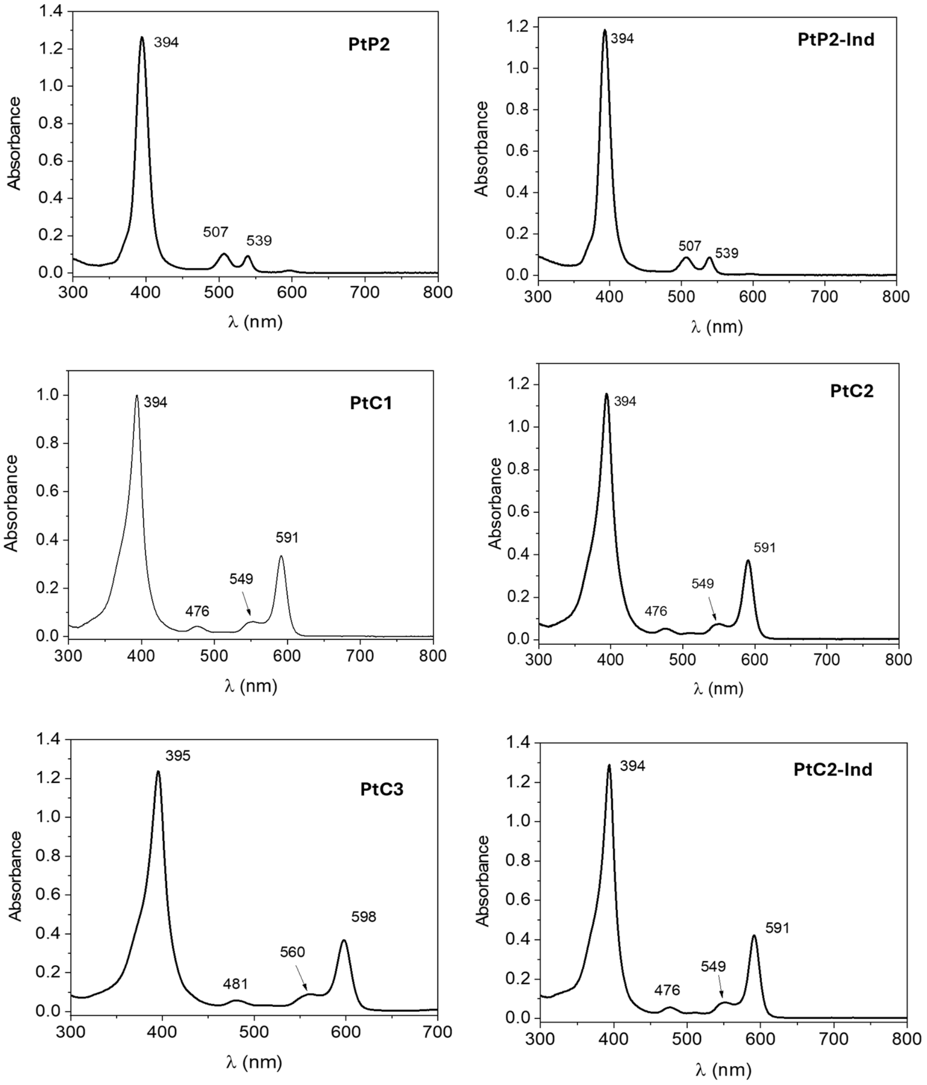

Figure S1. Absorption spectra of the platinum complexes (**PtP2**, **PtP2-Ind**, **PtC1**, **PtC2**, **PtC3** and **PtC2-Ind**) in DMF.

2- NMR spectra

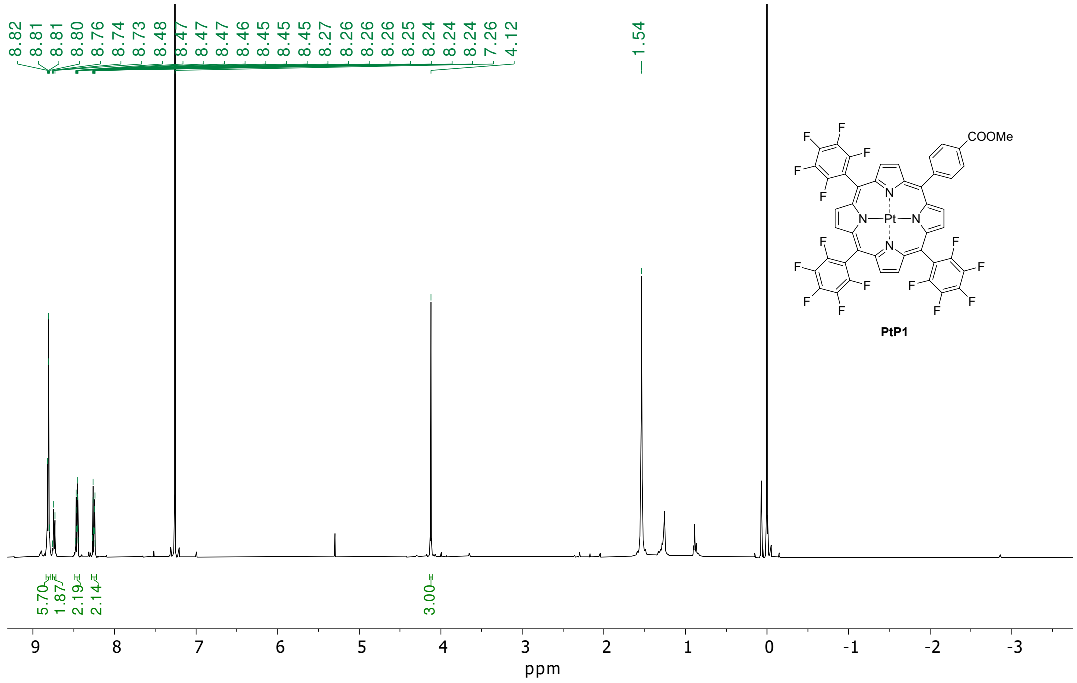

Figure S2. ^{1}H NMR (400.14 MHz, $CDCl_3$) spectrum of **PtP1**.

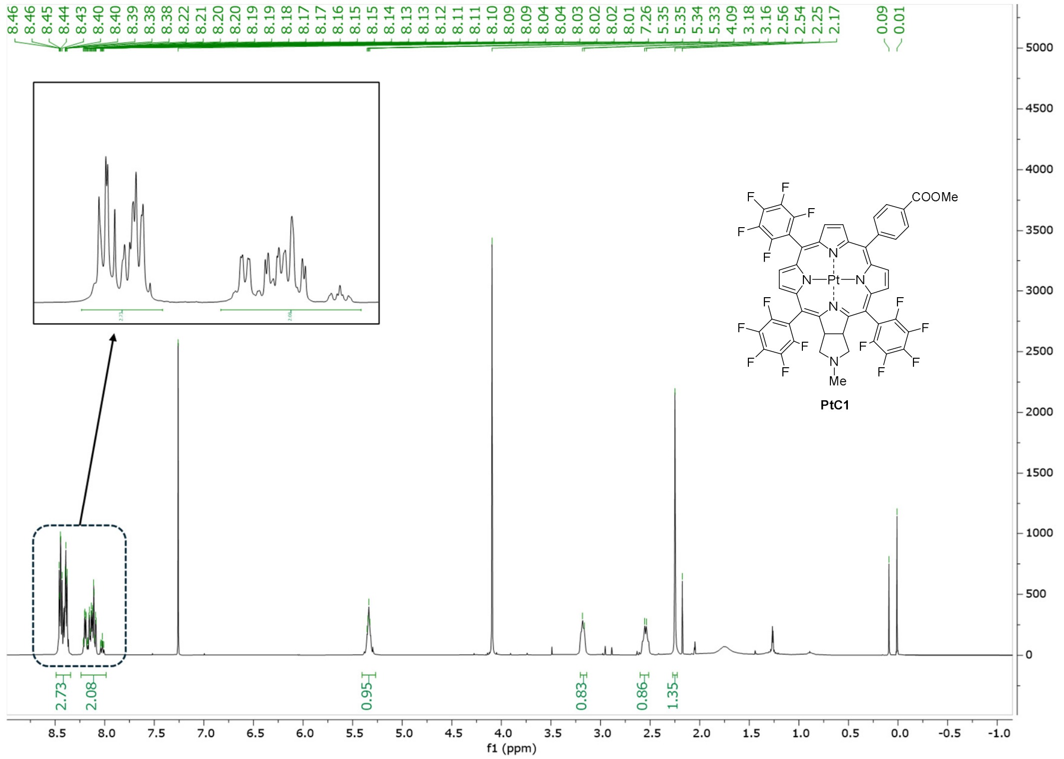

Figure S3. ^{1}H NMR (400.14 MHz, $CDCl_3$) spectrum of **PtC1**.

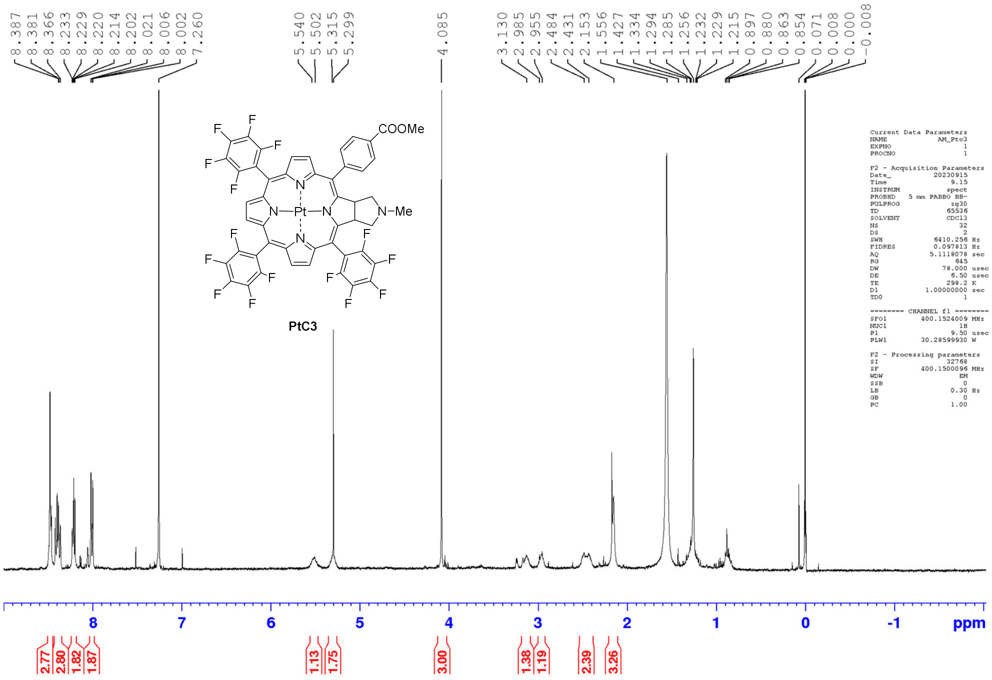

Figure S4. ^{1}H NMR (400.14 MHz, $CDCl_3$) spectrum of **PtC3**.

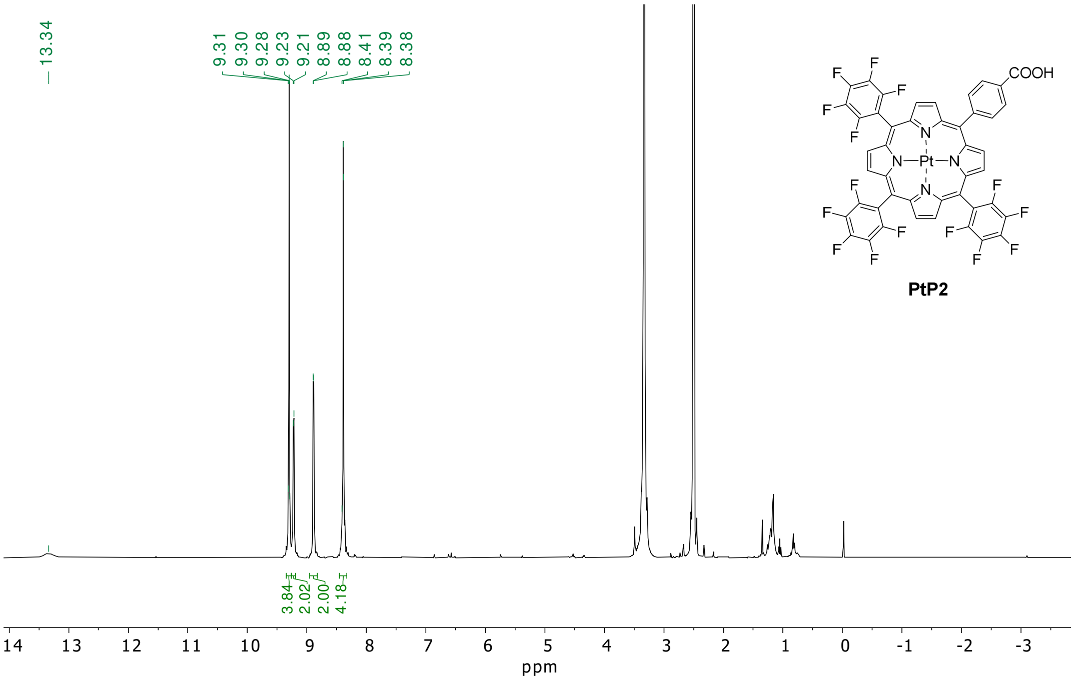

Figure S5. ^{1}H NMR (400.14 MHz, DMSO-d_6) spectrum of **PtP2**.

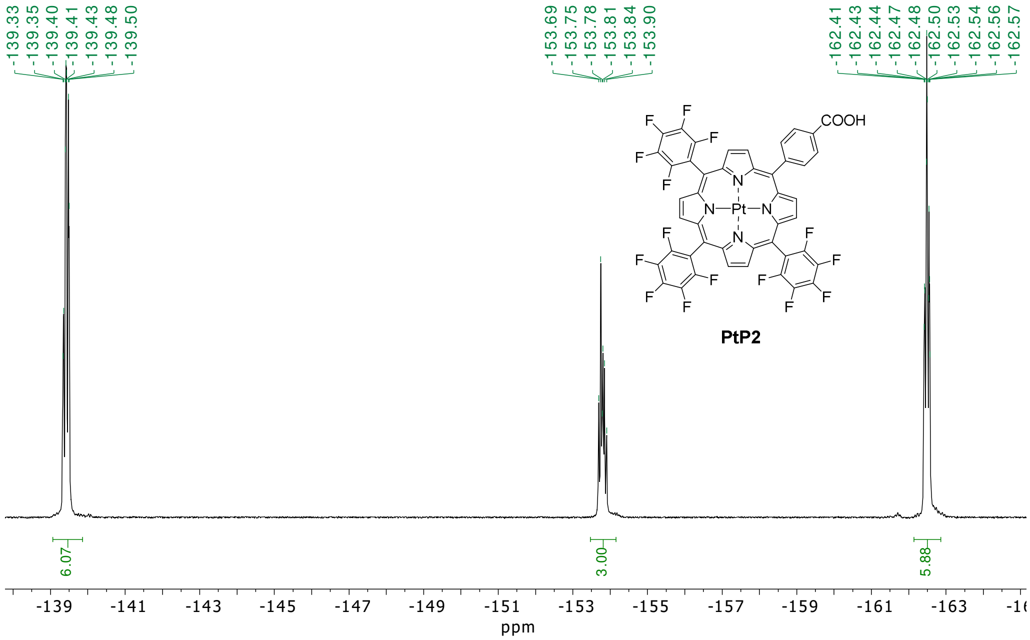

Figure S6. ^{19}F NMR (376.46 MHz, DMSO-d$_6$) spectrum of **PtP2**.

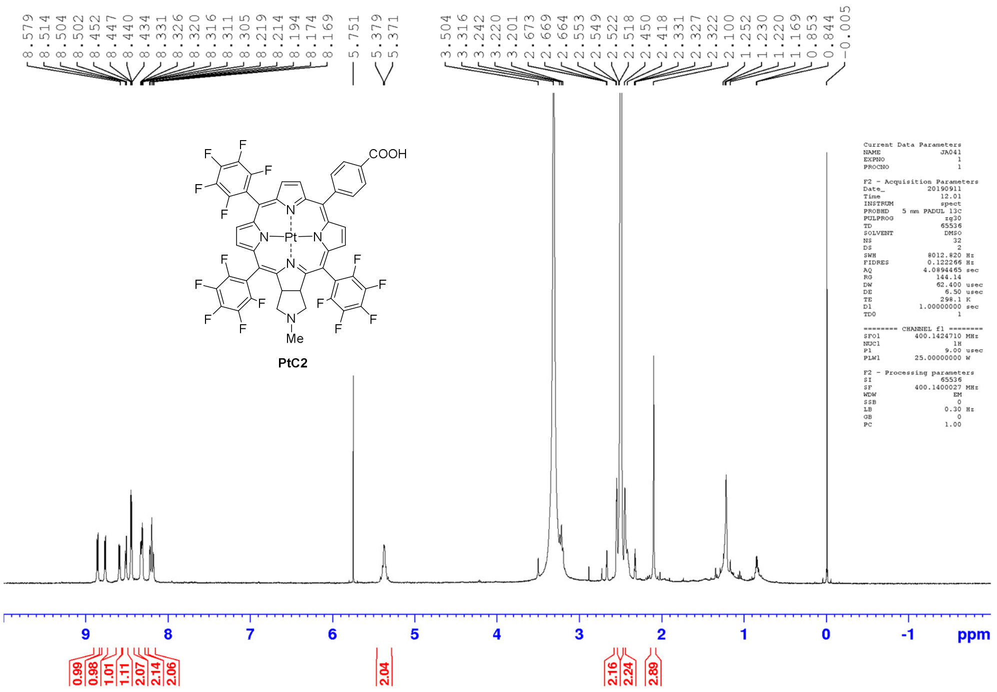

Figure S7. ^{1}H NMR (400.14 MHz, DMSO-d$_6$) spectrum of **PtC2**.

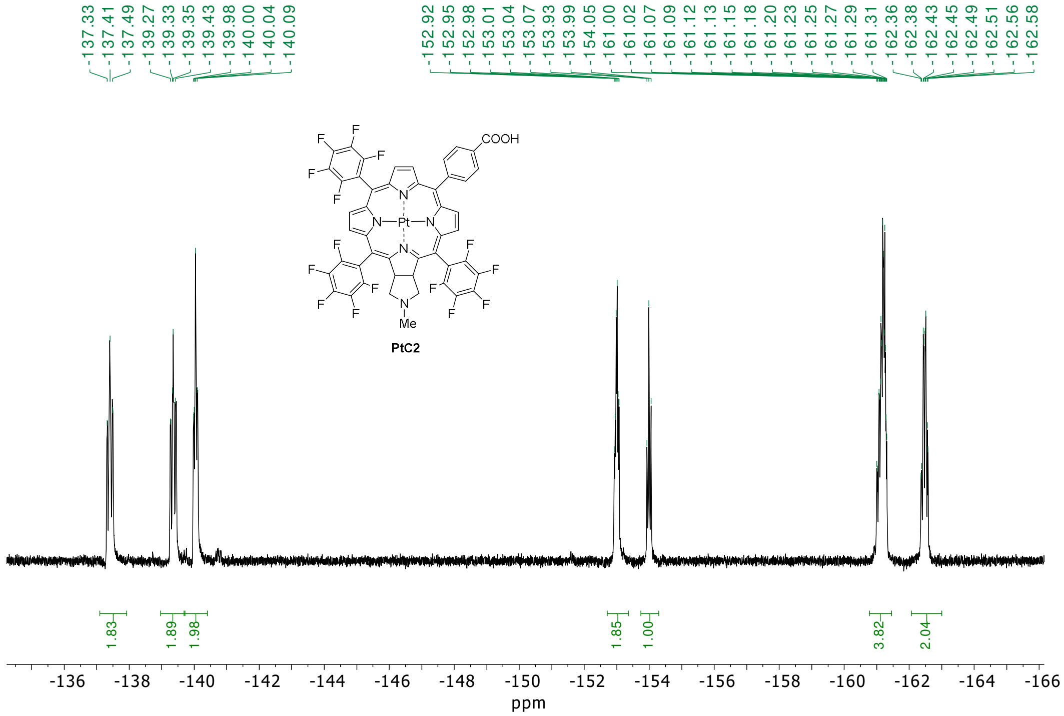

Figure S8. ^{19}F NMR (376.46 MHz, DMSO-d$_6$) spectrum of **PtC2**.

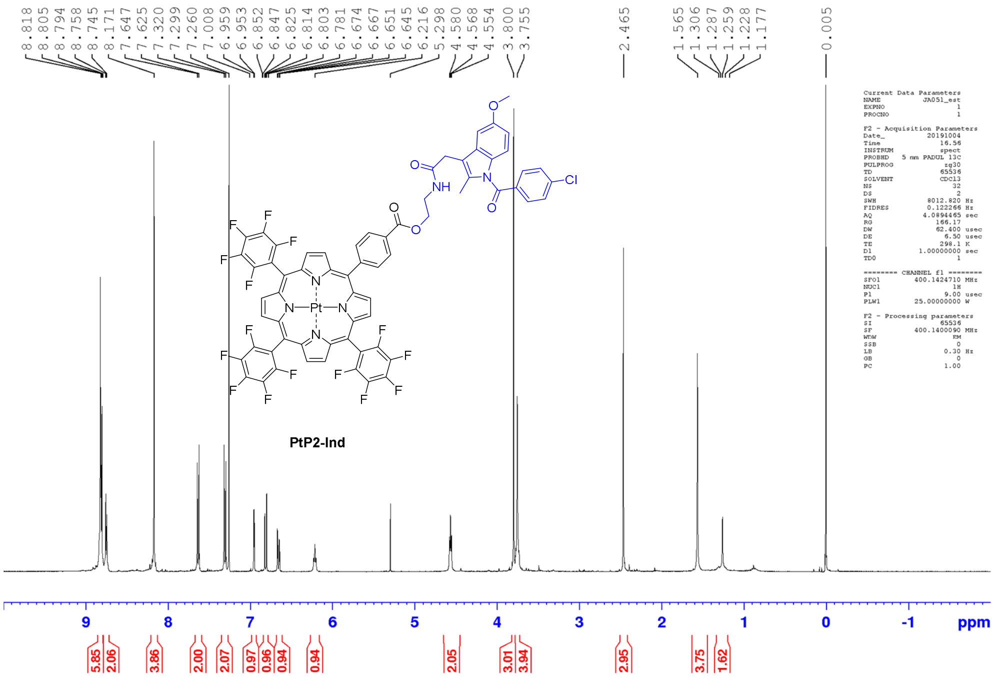

Figure S9. ^{1}H NMR (400.14 MHz, $CDCl_3$) spectrum of **PtP2–Ind**.

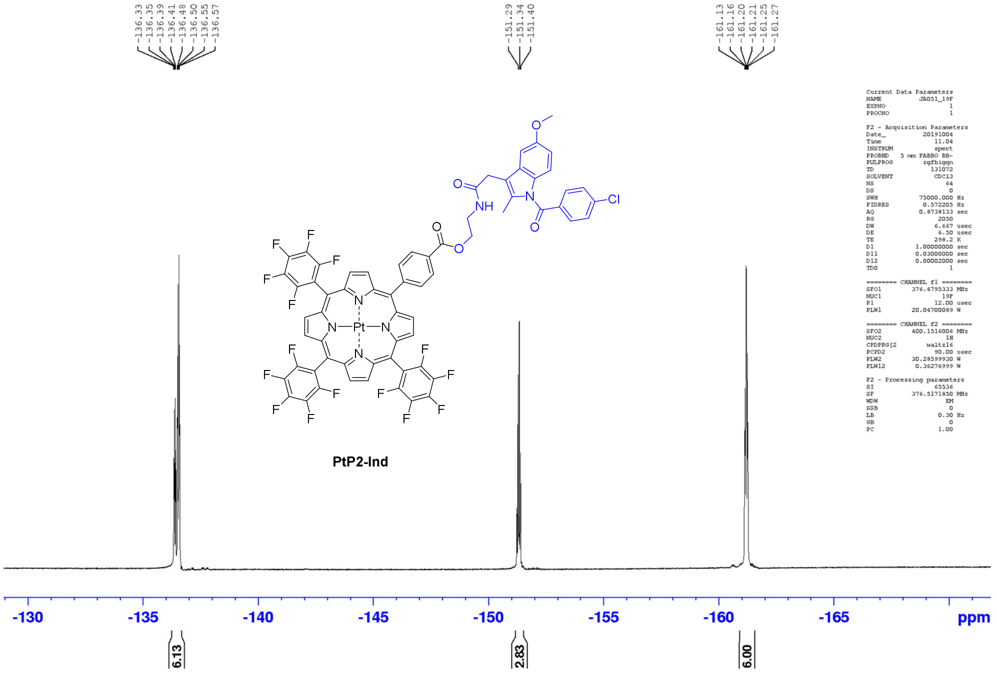

Figure S10. ^{19}F NMR (376.46 MHz, $CDCl_3$) spectrum of **PtP2–Ind**.

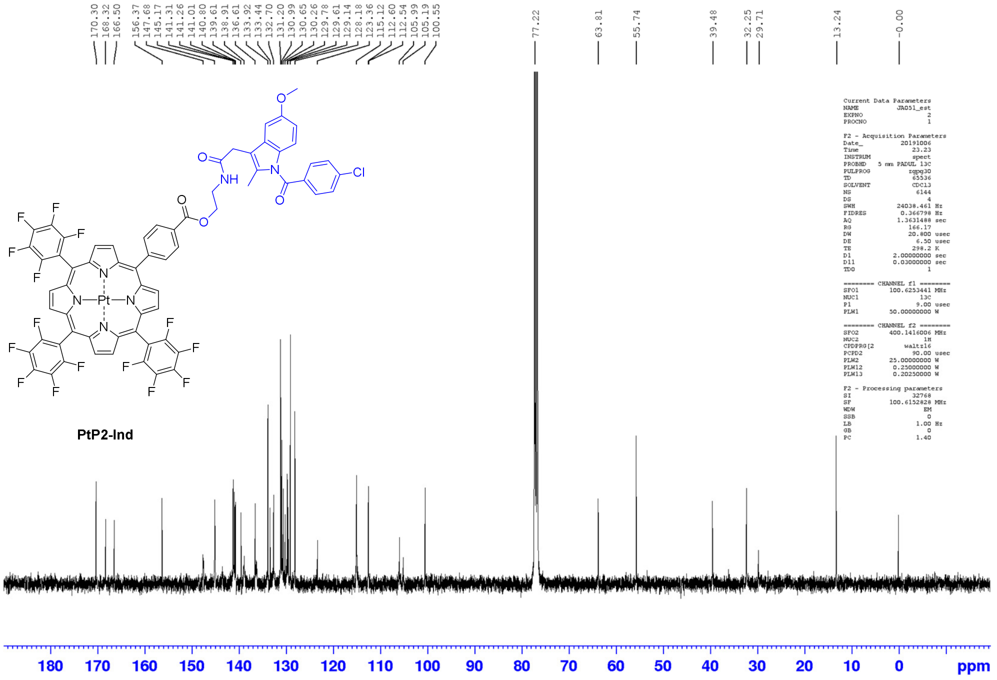

Figure S11. ^{13}C NMR (100.63 MHz, $CDCl_3$) spectrum of **PtP2–Ind**.

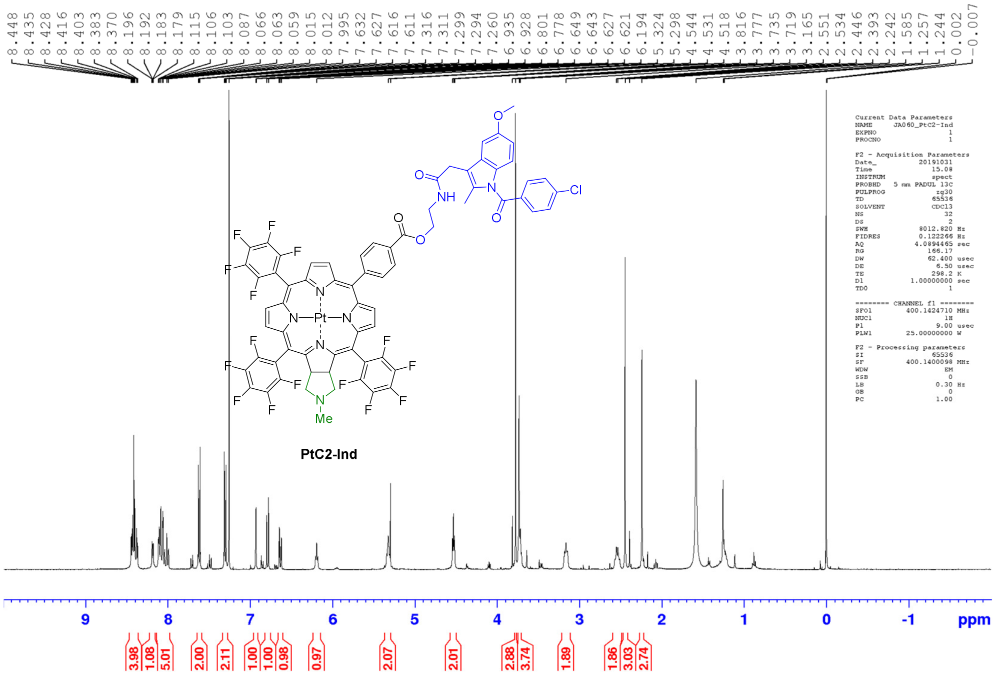

Figure S12. ^{1}H NMR (400.14 MHz, $CDCl_3$) spectrum of **PtC2–Ind**.

3- Mass spectra

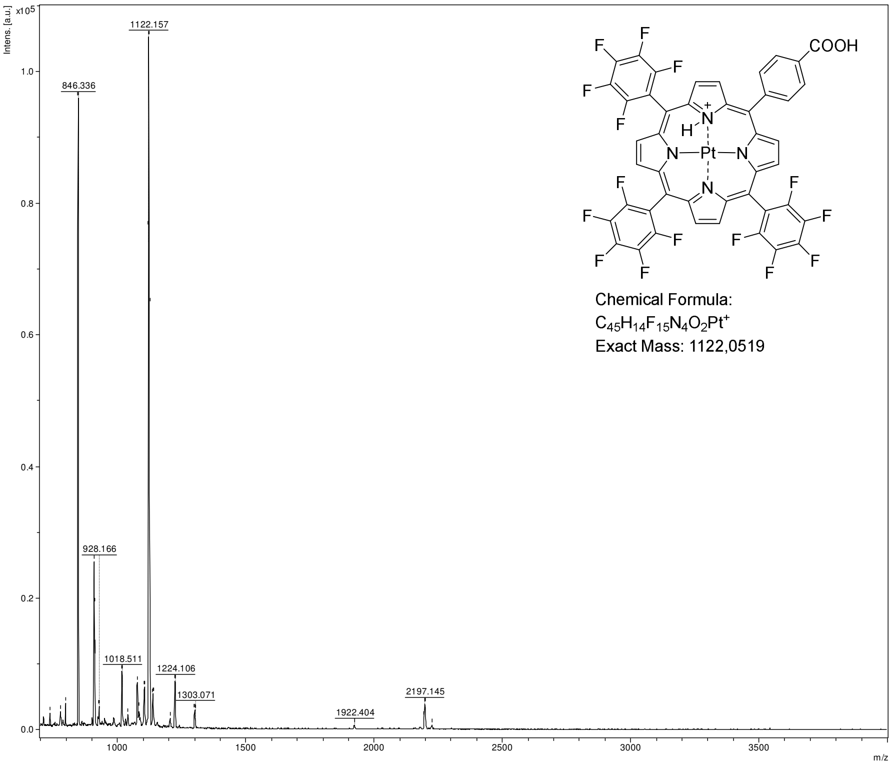

Figure S13. MS (MALDI-TOF) spectrum of **PtP2**.

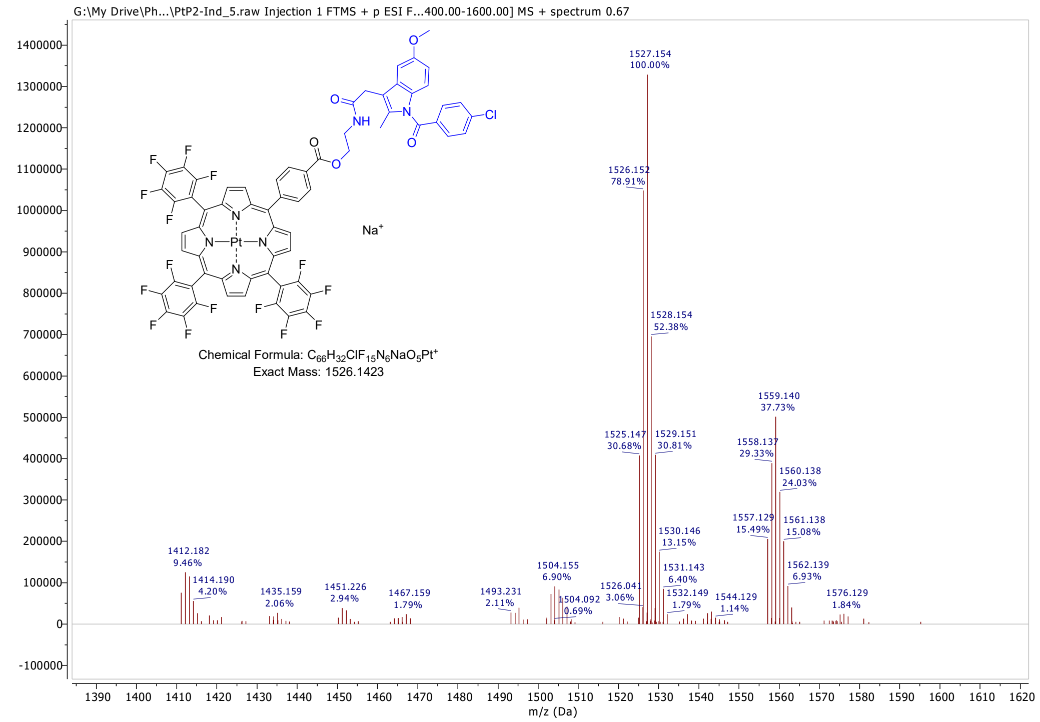

Figure S14. MS (ESI) spectrum of **PtP2-Ind**.

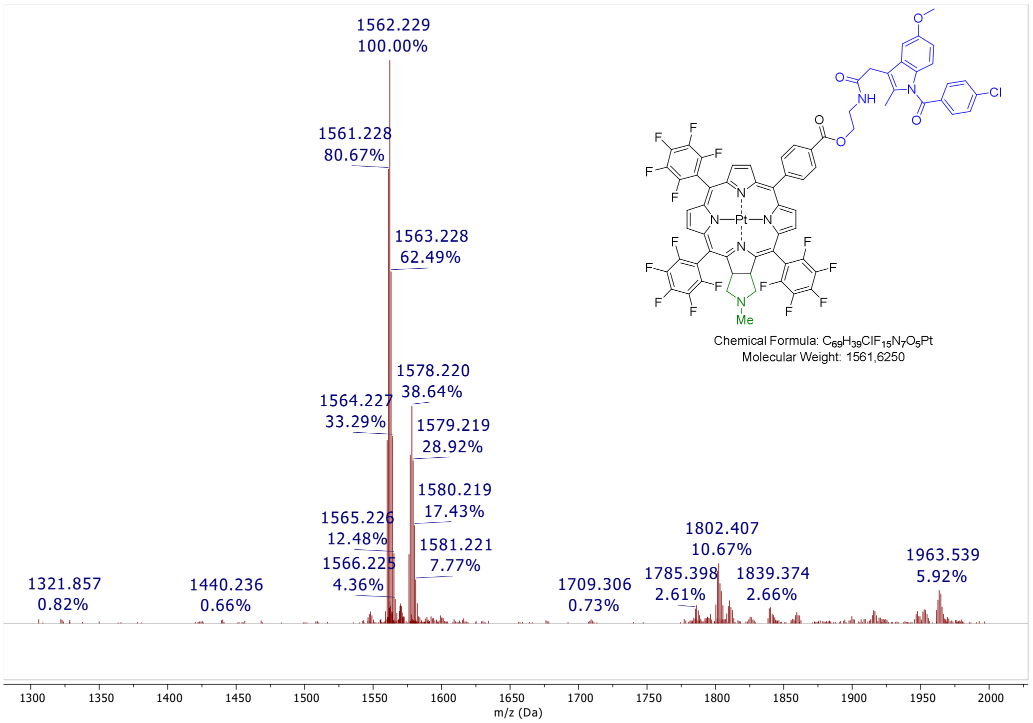

Figure S15. MS (ESI) spectrum of **PtC2-Ind**.

4- DFT Calculations

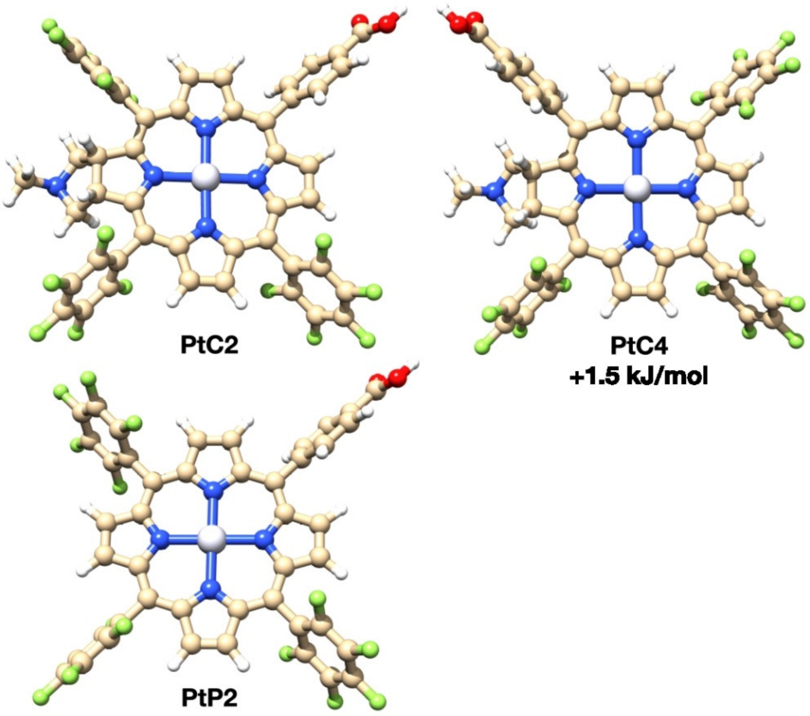

Figure S16. Structures of **PtC2**, **PtC4** and **PtP2** obtained by full geometry optimization by DFT calculations. The relative standard Gibbs free energy between the **PtC2** and **PtC4** isomers is also shown.

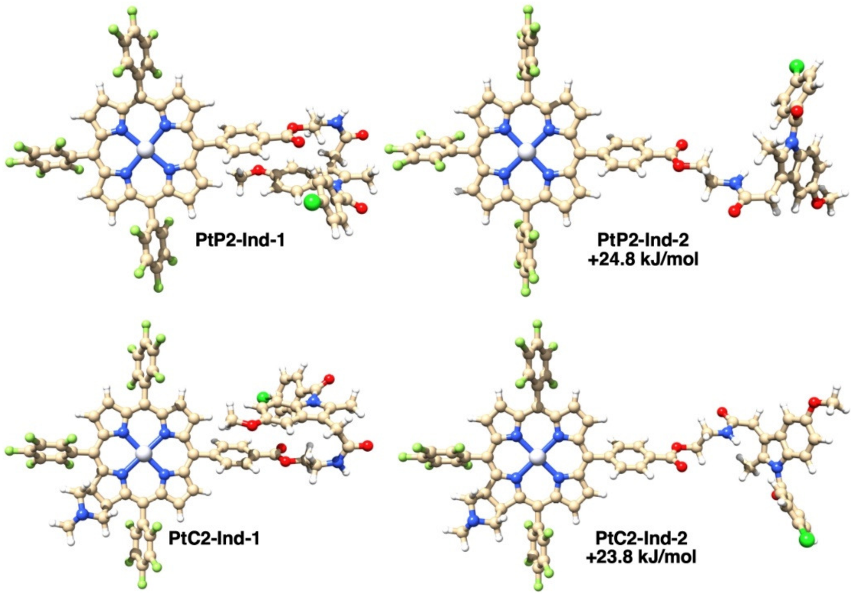

Figure S17. Structures of two conformations of **PtP2-Ind** and of **PtC2-Ind** obtained by full geometry optimization by DFT calculations. The relative standard Gibbs free energy of the two conformations (self-stacked and unfolded) is also shown.

5- X-ray

Table S2- Crystal and structure refinement data for **PtP1** structure.

Chemical formula	$C_{46}H_{14}F_{15}N_4O_2Pt$
M_r	1135.71
Crystal description	Red Prism
Crystal size /mm	0.35 × 0.12 × 0.10
Crystal system, space group	Monoclinic, *C2/c*
a /Å	8.5627(4)
b /Å	28.7767(16)
c /Å	35.495(2)
β /°	91.396(2)
Volume /Å^3	8743.6(8)
Z	8
$\rho_{calculated}$ /g cm^{-3}	1.726
F(000)	4384
μ /mm^{-1}	3.317
θ range /°	2.229 to 29.192
Index ranges	$-10 \le h \le 11$<br>$-39 \le k \le 39$<br>$-48 \le l \le 48$
Reflections collected	176101
Independent reflections	11762 (R_{int} = 0.0418)
Final R indices [I>2σ(I)]	R_1 = 0.0255<br>wR_2 = 0.0652
Final R indices (all data)	R_1 = 0.0323<br>wR_2 = 0.0738
$\Delta\rho_{max}$ and $\Delta\rho_{min}$ /e.Å^{-3}	1.523 and −1.195

6- Singlet oxygen quantum yield details

The ΦΔ values were determined by using 9,10-dimethylanthracene (DMA) as a chemical probe for singlet oxygen in air-saturated solutions, following established protocols.[1] The decrease in absorbance at 378 nm was monitored over time upon photoirradiation in the presence of each photosensitizer. The slope of the linear fit of the absorbance decay (A/A_0 vs. time) was used to evaluate the relative ΦΔ, using tetraphenylporphyrin (TPP, ΦΔ = 0.65 in DMF) as a reference compound. The ΦΔ for each compound was calculated by comparing the slopes according to the following equation:

$$\Phi_{\Delta}^{sample} = \Phi_{\Delta}^{TPP} \times (\frac{slope_{sample}}{slope_{TPP}}) \times (\frac{A_{TPP}}{A_{sample}})$$

Where Φ_{Δ}^{sample} is the singlet oxygen quantum yield of the sample, Φ_{Δ}^{TPP} is the singlet oxygen quantum yield of the standard (Φ_{Δ}^{TPP} = 0.65 in air-saturated DMF), $slope_{sample}$ is the slope of the absorbance decay of DMA at 378 nm in the presence of the sample, $slope_{TPP}$ is the slope of the absorbance decay of DMA at 378 nm in the presence of TPP, A_{TPP} is the absorbance of TPP at the irradiation wavelength and A_{sample} is the absorbance of the sample at the irradiation wavelength.

7- References

[1] J. C. J. M. D. S. Menezes, M. A. F. Faustino, K. T. de Oliveira, M. P. Uliana, V. F. Ferreira, S. Hackbarth, B. Röder, T. Teixeira Tasso, T. Furuyama, N. Kobayashi, A. M. S. Silva, M. G. P. M. S. Neves, J. A. S. Cavaleiro, *Chem. Eur. J.* **2014**, *20*, 13644-13655.
